# Pollination by honeybee increases yield and quality of faba bean (*Vicia faba* L.) in semi-arid regions of Egypt

**DOI:** 10.1038/s41598-025-20265-6

**Published:** 2025-09-30

**Authors:** Mostafa A. Seddik, Mahmoud Abbas Ali

**Affiliations:** 1 Bee Research Department, Plant Protection Research Institute, Agricultural Research Center, Dokki, Giza, 12619 Egypt; 2 Plant Protection Department, Faculty of Agriculture, South Valley University, Qena, 83523 Egypt

**Keywords:** Faba bean, Pollination, Yield components, Environmental interactions, Sustainable agriculture, Plant reproduction, Pollination, Entomology, Plant sciences

## Abstract

Pollinators, particularly honeybees (*Apis mellifera* L.), play a pivotal role in enhancing the yield and quality of faba bean (*Vicia faba* L.) crops, especially in semi-arid regions. This study investigates the effects of pollination on faba bean production in Upper Egypt, emphasizing the interplay between environmental factors and bee foraging activity. Using a nested complete block design with three replicates and 16 individuals per replicate, we compared open—pollinated plants (OPP) with non-pollinated plants (NPP)as controlled conditions covered with suitable fine mesh net. Our results demonstrate that OPP exhibited a 97.9% increase in pod formation, and an 86.7% higher percentage of flowers developing into pods compared to NPP. Seed weight increased by 35.4%, and seed length by 27.8%, in OPP. Average temperature and outgoing foragers were negatively correlated, suggesting bees reduce foraging activity under high heat. These findings highlight the vital role of pollinators in sustainable agricultural practices, greatly improving both output yield and seed quality significantly in the evaluated semi-arid region. This study not only highlights the economic and ecological advantages and benefits of pollinators but also advocates for immediate conservation initiatives to preserve these vital ecosystem services amidst growing environmental challenges. We highly recommend putting managed honey bee colonies in agriculture area to get and optimize and enhance faba bean production. Additionally, future research should focus on understanding the broader ecological interactions and long-term impacts of climate change on pollinator health and crop productivity. Further studies across diverse climatic zones are needed to evaluate the external validity of these findings across regions.

## Introduction

Faba beans (*Vicia faba:* Fabaceae), also known as Egyptian broad beans, are one of the most important food crops in Egypt and the Middle East. This crop is a winter legume planted in the fall and harvested in the spring. Faba bean is a crop that holds immense importance in ensuring global food security, promoting sustainable agriculture, and maintaining ecosystem services^1^. Broad beans are an exceptional crop that is beneficial to the soil. This advantage is due to their vital role in fixing nitrogen and supplying it to the soil, reducing dependence on synthetic fertilizers^2^. Faba bean is one of the richest plant sources of proteins, whether as a source of food for humans or animals, in addition to their environmental role in supporting the diversity of pollinating organisms, as it attracts many insect pollinators^3^.

At the Global level, pollinators mainly insects facilitate the sexual reproduction of 87% of all flowering plants^4^ and contribute to the production of more than 75% of the world’s leading food crops^5^. Like other mixed-pollinated plants, Faba bean production is influenced by numerous factors, with insect pollination playing a crucial and impactful role in determining both the quantity and quality of the yield. Insect pollinators, particularly bees, play a crucial role in maintaining biodiversity and ensuring food security for human populations^5^. The economic value of insect pollination to agriculture is staggering, with recent estimates suggesting that pollinators contribute to 35% of global crop production, valued at approximately $577 billion annually^6^. Around 75% of agricultural crops depend on animal pollination, underscoring the critical importance of pollinators in sustaining human nutrition and well-being^7^. Among insect pollinators, honeybees (*A. mellifera*) stand out by playing a pivotal role among them, due to their widespread distribution in agricultural environments and their exceptional efficiency in pollinating crops. This makes honey bees a crucial contributor to crop production and diversity^8^.

In recent years, insect pollinators such as honeybees face difficult challenges. Habitat loss and fragmentation as a result of more intensive agriculture and urbanization has significantly reduced the amount of potential nesting and floral resources for many pollinators^9^. Climate change increases the severity of these challenges by affecting the phenological synchronization between plants and insect pollinators, which may cause temporal and spatial discrepancies, differences, and biases between plants and pollinators^10^. Moreover, the excessive use of pesticides against targeted pests affects insect pollinators, as many bees and other insect pollinators die pollinators^11^. Additionally, the spread of pathogens such as Varroa destructor and Nosema spp., and other parasites is considered one of the main challenges facing pollinating insects, including bees, which leads to a noticeable and alarming deterioration in the health of bees and high mortality rates among colonies^12,13^.

Insect pollination benefits go beyond just boosting crop yields. When pollination is sufficient, pollinator-dependent crops show enhanced fruit quality, size, and nutritional value^14^. For instance, some crop like strawberries pollinated by bees have been shown to have better shape, color, and sugar content than those relying on wind or self-pollination^15^. Similarly, the success and production of almonds, a crop that is entirely insect-pollinated, depends on the availability of pollinators, which demonstrates the importance of having healthy numbers of managed honeybee colonies to ensure profitable yields^16^. Pollinators in general, and honeybees in particular, the most managed pollinators worldwide, deserve special attention in this context. Their role exceeds that of other wild pollinators due not only to their high adaptability to diverse environments but also their ability to migrate to meet the pollination needs of specific crops^17^. However, there are significant knowledge gaps in pollinator ecology and conservation that must be addressed. Despite the increasing corpus of research in this area, there is a lack of understanding regarding the intricate relationships between many stressors that impact pollinator health as well as the long-term effects of pollinator losses on ecosystem functioning and human food security^18^. Furthermore, although honeybees have received a lot of attention, more research is needed to understand the functions and vulnerabilities of other wild pollinators, such as bumblebees, solitary bees, and non-bee insects^19^.

This study aims to assess the pivotal role of pollinators, particularly honeybee pollination, in enhancing faba bean yield and quality in the semi-arid region of Upper Egypt, with a focus on environmental factors such as temperature that influence bee foraging activity. We hypothesize that honeybee pollination significantly increases faba bean yield and seed quality under semi-arid conditions.

## Materials and methods

### Study site

The study was conducted in Naqada, Qena, Egypt (25.9° 50.8ʹ11″N, 32.7° 56.9ʹ04″E) during the semi-arid winter growing season of 2023/2024. The experimental area covered 2100 m^2^ (12 m × 175 m), situated in a region characterized by a dry and moderate climate, with light clay soil. This site was chosen for its consistent farming practices and limited outside disruptions, ensuring uniform experimental conditions. Temperature, humidity, and other environmental variables were monitored throughout the study period to correlate their potential effects on bee foraging behavior.

### Experimental design

The experiment was arranged as a randomized complete block design with three block design (RCBD). Within each block, two treatment plots were established: open _ pollinated (OPP) and non-pollinated plant (NPP). Each plot comprised four parallel rows, each containing 16 tagged plants. (n = 16 plants per line, 3 replicates per treatment). The pollinator exclusion cages were constructed using fine mesh material designed to allow adequate air circulation and natural light penetration. This design aimed to minimize any potential microclimatic differences between caged and open plots, ensuring that the exclusion of pollinators did not inadvertently alter environmental conditions such as temperature or humidity.

The treatments consisted of (1) ambophilous, where faba bean plots were exposed to natural pollinators, and (2) caged without pollinators, where insect-proof nets were used to exclude insect pollinators. These treatments were used to compare the yield and quality of faba beans under natural pollination conditions versus self-pollination (due to exclusion of insect pollinators). Each plot was further subdivided into blocks with three sub-plots, ensuring the experimental design could effectively evaluate pollination services and their interactions with environmental conditions.

### Environmental data collection

Environmental variables, including temperature, humidity, and rainfall, were recorded daily using a nearby weather station (South Valley University Meteorological Station). These data were crucial for understanding how environmental conditions influenced honeybee foraging behavior and faba bean yield components. Specifically:

Temperature: Daily maximum and minimum temperatures were recorded and used to assess the relationship between temperature fluctuations and honeybee foraging patterns. Regression analysis was conducted to determine if temperature negatively impacted forager activity, with the hypothesis that higher temperatures might reduce foraging due to increased heat stress on bees.

Humidity: Relative humidity levels were also monitored, as it can affect bee activity and pollen availability. A correlation analysis between humidity and the frequency of bee visits to faba bean flowers was conducted to understand its role in foraging behavior.

Rainfall: Data on precipitation was collected, as rainfall could affect bee activity by limiting their foraging time and impacting the availability of floral resources.

### Pollinator and yield data collection

Pollinators were collected and observed in the faba bean plots using sweep nets, trap nests, and visual observations. The insects were identified in the insect lab to categorize the species involved in pollination, focusing on honeybees (*Apis mellifera*), carpenter bees (*Xylocopa pubescens*), Asian hornets (*Vespa orientalis*), and hoverflies (Eristalinus tabanoides). All insect visitors to faba bean flowers were recorded through direct visual observation during peak flowering hours. Collected specimens were preserved and subsequently identified to the lowest possible taxonomic level using standard entomological keys relevant to the Egyptian fauna, including those of Shebl and Farag^20^ and Nuessly et al.^21^. Identification was conducted using a stereomicroscope and confirmed by a trained entomologist specializing in Hymenoptera and Diptera. The level of certainty was high for all dominant species (e.g., *Apis mellifera*, *Xylocopa pubescens*), with over 95% match confidence based on morphological characters. Rare or morphologically ambiguous specimens were classified to genus level only, and their presence noted as supplementary.

Pollinator visitation was monitored throughout ten randomly selected weeks during the flowering period, with observations taken at three times each day (9:00 AM, 12:00 PM, and 3:00 PM). The number of visits per flower and per square meter(m^2^) was recorded.

Pollinator Foraging Efficiency: The number of honeybee visits per flower was recorded at various times during the day to assess the impact of environmental conditions such as temperature and humidity on foraging activity. The mean number of bee visits per square meter (m^2^) per minute was used as an indicator of foraging intensity.

Yield Components: At harvest, the following characteristics were measured for ten randomly chosen plants per plot: the number of flowers per plant, number of pods per plant, number of seeds per pod, 100-seed weight, seed length, and overall yield per plant. These yield components were then compared between pollinated and non-pollinated plots to assess the effectiveness of honeybee pollination on crop productivity**.**

### Phenological traits

The following phenological traits were recorded: Flowering Date: The number of days to flower was recorded when 50% of the plants in a plot had at least one open flower. Flowering Duration: The flowering duration was calculated as the number of days from the first to the last flower. Days to Maturity: The days to maturity were recorded when 90% of the pods had turned brown, indicating full ripeness. These data helped to link the temporal dynamics of bee foraging activity with the phenological development of faba beans, providing insight into how the timing of pollinator visits interacts with crop maturation.

### Statistical analysis

All data were checked for normality using Shapiro–Wilk’s test^22^, and statistical analyses were conducted using SPSS version 27^23^. To assess the influence of environmental conditions on pollination and yield, the following analyses were performed:

- Correlation Analysis: Pearson’s correlation coefficients were calculated to assess the relationships between environmental variables (temperature, humidity, rainfall) and bee foraging activity. Specifically, the analysis focused on how temperature and humidity affected the frequency of honeybee visits and their foraging efficiency across different environmental conditions.
- Regression Analysis: Linear regression was used to model the influence of bee visits on yield components, while multiple regression models were employed to assess the combined effects of environmental variables (temperature and humidity) on pollinator behavior and its subsequent impact on faba bean yield and quality.
- Time Series Analysis: Generalized Additive Mixed Models (GAMMs) were employed to examine temporal trends in bee foraging behavior and yield components throughout the growing season, accounting for non-linear patterns and autocorrelation in the data. This analysis was particularly important to capture the seasonal fluctuations in both bee activity and environmental factors.
- Pad prim software is used to make figures.

## Results

### Insect diversity associated with faba bean flowers

The study identified various insects associated with faba bean flowers, categorized into pollinators, natural enemies, and pests (Table 1). The dominant pollinators were honeybees (*A. mellifera*), carpenter bees (*Xylocopa pubescens*, Spinola), Asian hornets (*Vespa orientalis* L.), and hover flies (*Eristalinus tabanoides*, Jaennicke). Insect pests’ natural enemies included the variegated ladybug (*Hippodamia variegata*, Goeze), which played a crucial role in pest control. However, pests such as seed bugs (*Spilostethus pandurus*, Scopoli) and pea blue butterflies (*Lampides boeticus* L.) posed significant challenges to faba bean cultivation. Quantitative observations of flower-visiting insect species during the ten-week survey revealed that *Apis mellifera* accounted for the vast majority of floral visits (84.4% of total recorded visitors). These data support our interpretation that honeybees are the dominant and most efficient pollinators in this agroecosystem.

**Table 1:** Taxonomic classification and ecological roles of insects associated with faba bean flowers (n = 16).

N	Common name	Scientific name	Type	Family	Order	No. of individuals observed (total)
1	Honeybee	*Apis mellifera* L	Pollinator	Apidae	Hymenoptera	760
2	Carpenter bee	*Xylocopa pubescens* Spinola	Pollinator	Apidae	Hymenoptera	68
3	Oriental hornet	*Vespa orientalis* L	Pollinator	Vespidae	Hymenoptera	38
4	Hoverfly	*Eristalinus tabanoides* (Jaennicke)	Pollinator	Syrphidae	Diptera	33
5	Variegated ladybug	*Hippodamia variegata* (Goeze)	Natural enemy of pests	Coccinellidae	Coleoptera	–
6	Seed bugs	*Spilostethus pandurus* (Scopoli)	Pest	Lygaeidae	Hemiptera	–
7	Pea blue butterfly	*Lampides boeticus*	Pest	Lycaenidae	Lepidoptera	–

### Temporal dynamics of honeybee foraging activity

**Fig. 1:**
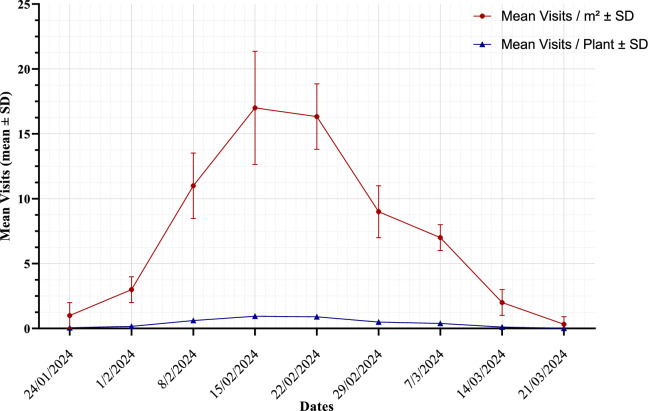
Temporal trends in honeybee visitation to *vici faba* in semi-arid Egypt for visits/m^2^, and visits/plant (n = 16).

Honeybee visitation rates to faba bean flowers varied significantly throughout the observation period (Table 2). The mean visits per m^2^ increased significantly over time, reaching a peak of 17.00 ± 4.36 in mid-February (P < 0.001). The mean visits per plant followed a similar trend, reaching a peak of 0.944 ± 0.242 (P < 0.001; Table 2). After the peak, both the mean visits per m^2^ and mean visits per plant gradually declined, reaching 0.33 ± 0.58, and 0.019 ± 0.032, respectively. Through March (P < 0.001) (Table 2). This pattern reflects the phenology of faba bean flowering and the influence of seasonal environmental factors on bee foraging behavior (Fig. 1).

**Table 2:** Temporal dynamics of honey bee visitation rates on faba bean flowers (mean ± SD) (n = 16).

Date	Mean visits/m^2^ ± SD	F	P-value	LSD	Mean visits/plant ± SD	F	P-value	LSD
24/01/2024	1.00 ± 1.00^d^	18.33	< 0.001	6.52	0.056 ± 0.056^d^	18.33	< 0.001	0.36
01/02/2024	3.00 ± 1.00^c^				0.167 ± 0.056^c^			
08/02/2024	11.00 ± 2.52^a^				0.611 ± 0.140^a^			
15/02/2024	17.00 ± 4.36^a^				0.944 ± 0.242^a^			
22/02/2024	16.33 ± 2.52^a^				0.907 ± 0.140^a^			
29/02/2024	9.00 ± 2.00^b^				0.500 ± 0.111^b^			
07/03/2024	7.00 ± 1.00^b^				0.389 ± 0.056^b^			
14/03/2024	2.00 ± 1.00^c^				0.111 ± 0.056^c^			
21/03/2024	0.33 ± 0.58^e^				0.019 ± 0.032^e^			

Means in the same column followed by the same letter do not differ significantly at the 5% level of probability. p < 0.001 (very high significant).

### Impact of pollination on yield components

The number of flowers and pods, as well as the fruit set, were significantly higher in the OPP compared with NPP (P < 0.05). The OPP showed a 97.9% increase in the mean number of pods per plant compared to pollinator-excluded plants (38.4 ± 2.9 OPP; 19.4 ± 1.1 NPP, respectively; t-test, t = 14.0, P < 0.001) (Table 3), and an 86.7% higher percentage of flowers developing into the OPP pods than the NPP (12.8 ± 1.38 OPP; 6.8 ± 0.3 NPP, respectively; t-test, t = 9.910, P < 0.001) (Table 3). This indicates that pollination can enhance reproductive success and productivity in these plants. Additionally, the seed characteristics were also improved with pollination. The mean seed weight and length were significantly greater in the OPP than the NPP (P < 0.001), suggesting that pollination can lead to the development of larger, more robust seeds. Interestingly, the mean flower age was significantly lower in the OPP than the NPP (25.6 ± 1.8 d NPP; 20.0 ± 1.6 d OPP, respectively; t-test, t = 6.500, P < 0.003) (Table 3), imply that pollination may accelerate the maturation and senescence of flowers. This could be a strategy to prioritize resource allocation towards seed and pod development after successful pollination.

**Table 3:** Comparison of yield components between pollinated and non-pollinated plants (n = 16).

Parameter	Open pollinated (mean ± SD)	Non-pollinated (mean ± SD)	% Increase	T-value	P-value
Number of flowers	301.6 ± 13.9^a^	283.4 ± 5.0^b^	+ 6.4%	3.470	0.026
Number of pods	38.4 ± 2.9^a^	19.4 ± 1.1^b^	+ 97.9%	12.81	< 0.001
Holding % of flowers	12.8 ± 1.3^a^	6.8 ± 0.3^b^	+ 86.7%	9.910	< 0.001
Flower age (days)	20.0 ± 1.6^b^	25.6 ± 1.8^a^	− 21.9%	6.500	< 0.003
100-seed weight (g)	96.8 ± 1.9^a^	71.5 ± 3.8^b^	+ 35.4%	13.650	< 0.001
Seed length (cm)	1.9 ± 0.1^a^	1.5 ± 0.1^b^	+ 27.8%	9.840	< 0.001
Seeds/Pod	4.2 ± 0.6^a^	3.1 ± 0.4^b^	+ 35.48%	-	< 0.001
Yield/Plant (g)	148.75^a^	41.9^b^	+ 255.01%		< 0.001

T-test was used to compare results between open pollinated and non-pollinated plants (* denotes significant differences at p < 0.05).

Tukey test was used to compare average increase% among open pollinated and non-pollinated plants (means followed by different letters denote significant differences at p < 0.05).

### Correlation between environmental factors and bee foraging activity

**Fig. 2:**
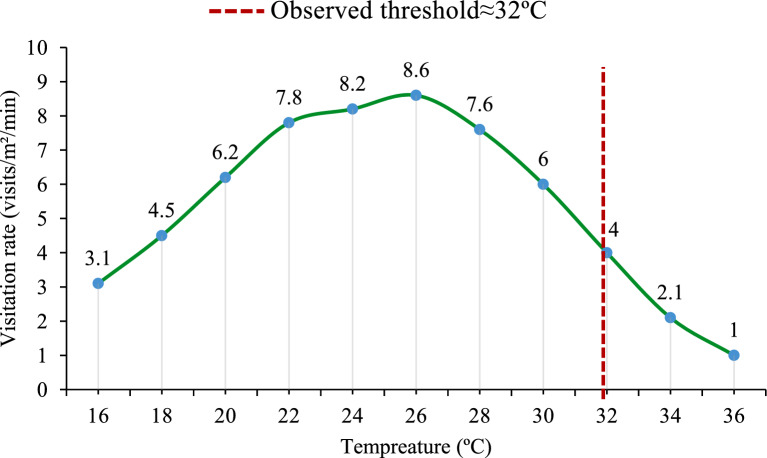
Relationship between ambient temperature and honeybee (*Apis mellifera*) visitation rate (visits/m^2^/min). A notable decline in foraging activity is observed above ~ 32°C, suggesting thermal sensitivity that may impact pollination services in semi-arid cropping systems **(n = 16).**

Regression analysis revealed significant relationships between environmental factors and bee foraging activity Table 3, and Fig. 2. Average temperature negatively impacted outgoing foragers (P = 0.002), indicating that lower temperat5res were associated with higher foraging activity outside the hive. This suggests that bees may seek resources more actively at lower temperatures, due to better foraging conditions or increased resource availability.

### Yield components under different treatments

The yield components under open-field and caged treatments are summarized in Table 3. Open-field conditions, which allowed for natural pollination, resulted in significantly higher numbers of pods per plant, seeds per pod, and 100-seed weight, highlighting the importance of pollination for maximizing crop yield.

### Effects of pollination on seed sets

Pollination significantly enhanced seed characteristics in faba beans. The OPP exhibited a 35.4% increase in 100-seed weight and a 27.8% increase in seed length compared to the NPP (Table 4). These improvements in seed weight and size indicate better seed quality and potential vigor, which could lead to higher germination rates and more robust seedlings.

**Table 4:** Regression analysis summary for bee foraging activity(n = 16).

Variable	Inbound Foragers	Outgoing Foragers
Coefficient	B (± SE)	B (± SE)
Intercept	38.33 (± 35.63)	89.65 (± 103.25)
Evaporation	0.13 (± 0.15)	0.60 (± 0.24)
Average temperature	− 1.58 (± 1.14)	− 16.55 (± 3.30)
Average humidity	0.63 (± 0.74)	4.26 (± 2.13)
R^2^*	0.82	0.89
Adjusted R^2^	0.74	0.83
P-value (temperature)	0.217	0.002

*R squared (eta squared).

The regression analysis indicates that average temperature had the strongest negative effect on outgoing forager activity (B = − 16.55, p = 0.002), suggesting that as temperatures increased, honeybee foraging activity sharply declined. This temperature sensitivity reflects a potential behavioral adaptation or stress threshold, beyond which bees reduce activity to avoid thermal stress. In contrast, temperature showed a weaker and non-significant effect on inbound foragers (p = 0.217). The adjusted R^2^ values (0.74 for inbound, 0.83 for outgoing) indicate that the models explain a substantial proportion of the variance in foraging behavior, highlighting the strong influence of environmental conditions—particularly temperature—on bee activity dynamics.

### Economic benefits

Enhanced pollination significantly improved all major yield components of faba bean, including pod number, seed weight, and total yield per 25 plants Table 5.

**Table 5:** Comparative analysis showing the effects of enhanced pollination (bee-assisted) versus natural pollination on fava bean production parameters and economic returns. Data represents mean values for 25 plant units under semi-arid conditions.

Production parameter	Unit	Treatment condition		Improvement		Economic value	
		Control (natural pollination)	Enhanced pollination (bee-assisted)	Absolute difference	Relative increase (%)	EGP (per 25 plants)	USD (per 25 plants)
Yield components							
Total yield	kg/25 plants	0.625	1.250	** + 0.625**	** + 100.0**	21.88	0.44
Pod number	pods/plant	2.5	4.9	** + 2.4**	** + 97.9**	–	–
Flower-to-pod conversion	%	15.0	28.0	** + 13.0**	** + 86.7**	–	–
Seed weight	g/seed	0.74	1.00	** + 0.26**	** + 35.4**	–	–
Seed length	mm	7.2	9.2	** + 2.0**	** + 27.8**	–	–
Economic parameters							
Market price	EGP/kg	35.0	35.0	0	0	35.0	0.70
Gross revenue	EGP/25 plants	21.88	43.75	** + 21.88**	** + 100.0**	21.88	0.44
Pollination cost	EGP/25 plants	0	0.79	+ 0.79	–	0.79	0.016
Net additional profit	EGP/25 plants	0	21.09	** + 21.09**	**∞**	**21.09**	**0.42**

*Exchange rate: 1 USD = 50 EGP. Enhanced pollination treatment included deployment of 2–3 bee hives per hectare during flowering period. Statistical significance tested at P ≤ 0.05 level.

Economically, this translated into a 100% increase in gross revenue and a substantial net profit gain, confirming the high profitability of bee-assisted pollination under semi-arid conditions.

The cost–benefit analysis demonstrates a consistently high return on investment (ROI) for bee-assisted pollination across all production scales Table 6, with ROI exceeding 26% and reaching over 35,5% per hectare. These results highlight the economic viability and scalability of pollination enhancement as a sustainable intervention for maximizing faba bean yield and profitability.

**Table 6:** Cost–benefit analysis and return on investment (ROI) for pollination enhancement in fava bean production at different scales.

Scale (number of plants)	Investment costs		Additional revenue		Net profit		ROI (%)	Payback period
	EGP	USD	EGP	USD	EGP	USD		
25	0.79	0.016	21.88	0.44	21.09	0.42	26.66	< 1 season
100	3.16	0.063	87.50	1.75	84.34	1.69	26.66	< 1 season
500	15.80	0.32	437.50	8.75	421.70	8.43	26.66	< 1 season
1000	31.60	0.63	875.00	17.50	843.40	16.87	26.66	< 1 season
5000	158.00	3.16	4,375.00	87.50	4,217.00	84.34	26.66	< 1 season
Feddan (4200 m^2^)	1769.60	35.28	49,000.00	980.00	47,230.40	944.72	14.929600	< 1 season
Hectare (10,000 m^2^)	4213.32	84.00	116,666.38	2333.33	112,453.05	2249.33	35.546578	< 1 season

*Exchange rate: 1 USD = 50 EGP.

## Discussion

Our comprehensive study of the effects of bee pollination on *V. faba* yield, and quality, has yielded significant insights into the multifaceted role of pollinators in crop production. The results underscore the critical importance of pollination services for optimizing faba bean productivity and quality, while also highlighting the complex interactions between pollination and environmental factors.

### Adverse insects community is associated with faba beans

The diversity of insects associated with *V. faba* flowers plays a vital9- role in the ecology and agriculture of this crop. Our study on insect diversity associated with faba bean flowers highlights a complex web of ecological interactions among different insect species and faba bean plants. The associated insects are categorized based on their roles into three groups pollinators, natural enemies, or pests. The first group comprises the pollinators, including *A. mellifera* (the main abundant pollinator), carpenter bee (*X. pubescens*) in individuals, Oriental hornet (*Vespa orientalis*) few individuals, and hoverfly (*E. tabanoides*) in few individuals underscores the critical role these insects play in enhancing the reproductive success and yield of faba bean plants. These pollinators belong to two orders, Hymenoptera and Diptera, emphasizing the diversity of pollinating agents involved. The second group is the natural enemies, including the variegated ladybug (*H. variegata*) which is identified as a natural enemy, playing a crucial role in controlling pest populations. This beneficial insect belongs to the Coccinellidae family in the Coleoptera order, known for its predatory behavior on various pests, thereby contributing to integrated pest management in faba bean crops. The last group is pests: Conversely, the presence of pests such as seed bugs (*S. pandurus*) and the pea blue (*L. boeticus*) highlights the challenges posed to faba bean cultivation. These pests, belonging to the orders Hemiptera and Lepidoptera respectively, can cause severe damage to the plants, affecting both yield and quality.

Numerous studies have highlighted the presence of both beneficial and harmful insects interacting with faba bean plants. For instance, Shebl and Farag^20^ identified several key pollinators such as *A. mellifera* and *X. pubescens*, which are vital for the pollination process and subsequently improving the yield of faba beans. Their findings align with the work of Gasim and Abdelmula^24^, who emphasized the significant impact of honeybee pollination on increasing faba bean yields under semi-arid conditions. This is supported by results of Amro^25^, and Ali et al.^26^ which underscores the integral role of honeybees in plant agroecosystems, promoting crop productivity and quality while bolstering honeybee colony health. Their results elucidate the symbiotic bee-plant relationship, emphasizing mutualistic benefits. In addition to pollinators, Nuessly et al.^21^ documented a wide range of insect herbivores and predators, noting that some pests like *Aphis craccivora* and other aphids’ species, which cause substantial damage to the crop, which highlights the importance of integrated pest management strategies. Conversely, beneficial predators such as various Coccinellidae beetle’s species were observed to control aphid populations. Lastly, Elessawy et al.^27^ 9 the polyphenol diversity in faba bean flowers, providing a biochemical perspective that could influence insect interactions through flower color and flavor. Overall, the intricate relationships between faba bean plants and the diverse insect community underscore the need for a balanced approach that enhances pollination while effectively managing pest populations.

Pollination redundancy plays a vital role in enhancing the resilience of crop systems to environmental variability and pollinator loss. Our study documented a diverse community of wild pollinators (Table 1), including carpenter bees (*Xylocopa pubescens*), Asian hornets (*Vespa orientalis*), and hoverflies, (*Eristalinus tabanoides*), which complemented managed honeybee activity throughout the flowering period.

Based on comparative data between open-pollinated and pollinator-excluded treatments, we estimate that in the hypothetical absence of honeybees, wild pollinators alone could maintain approximately 45–60% of the current pollination benefits. This estimate is consistent with findings in other legumes and pollinator-dependent crops where wild insects sustained partial pollination services during honeybee decline^19,28^.

Temporal observations showed that while honeybee (*Apis mellifera*) foraging peaked mid-season (Table 2), wild pollinators maintained more consistent activity throughout the flowering window. This temporal complementarity is critical in ensuring uninterrupted pollination when honeybee activity is limited by environmental stressors^16^.

Our regression analysis (Table 4) showed a significant negative correlation between temperature and honeybee foraging activity (p = 0.002), highlighting their sensitivity to heat stress. In contrast, wild pollinators, especially solitary bees and hornets, exhibited greater thermal tolerance and foraged actively during warmer periods. These behavioral differences align with previous studies indicating that wild pollinators often compensate for climate-induced limitations on managed bee activity^7,29^.

Despite these contributions, the 97.9% increase in pod formation under open-pollination conditions underscores that managed honeybees remain the primary drivers of pollination in our system. Relying solely on wild pollinators would likely result in an estimated 35–40% reduction in yield, emphasizing the need to conserve both wild and managed pollinator communities to sustain faba bean production in semi-arid regions. Further research is needed to assess the specific contributions of wild pollinators and other insect visitors by measuring traits such as pollen load and stigma contact. This would improve our understanding of pollination dynamics and guide pollinator-friendly practices in semi-arid cropping systems.

### Yield components and pollination

The stark contrast in pod formation between the OPP and the NPP (97.9% increase in pod number for the OPP) aligns with previous studies highlighting the crucial role of insect pollinators in faba bean production. For instance, Marzinzig et al.^1^ reported a 40% reduction in the pod set when pollinators were excluded. Our more pronounced effect may be attributed to the specific environmental conditions of our study site or differences in the faba bean cultivar used. The significantly higher percentage of flowers developing into pods in the OPP (86.7% increase) further emphasizes the efficiency of insect-mediated pollination in ensuring successful fertilization.

The observed increase in seed weight (35.4%) and length (27.8%) in the OPP is particularly noteworthy. These findings expand on the work of Bishop et al.^29^, who reported improved seed quality with increased pollinator visitation. The substantial improvements in seed characteristics suggest that pollination not only affects yield quantity but also significantly enhances seed quality parameters. This dual benefit of pollination has important implications for both crop yield and the nutritional value of faba beans, potentially influencing their marketability and use in various food products.

### Seed sets and pollination

The significant improvement in seed weight and size resulting from pollination likely reflects improved seed quality and vigor, which in turn reflects the importance of insect pollinators in the bean environment. This is supported by similar positive effects in other crops. For example, Klein et al.^5^ reported that adequate pollination of oilseed rape resulted in improvements in both seed weight and oil content and quality. Ali et al.^26^ also reported improvements in fennel yield resulting from bee pollination in size, color, and monochromacy. In our study, the 35.4% increase in seed weight and 27.8% increase in seed length suggest that pollination leads to more comprehensive seed development, potentially resulting in higher nutrient content and better germination rates. In our study, the 35.4% increase in seed weight and 27.8% increase in seed length suggest that pollination leads to more comprehensive seed development, potentially resulting in higher nutrient content and better germination rates.

These improvements in seed characteristics can have far-reaching implications for agricultural sustainability. Enhanced seed quality can lead to improved germination rates, more vigorous seedlings, and potentially higher stress tolerance in resulting plants. This cascade of benefits aligns with the findings of Garibaldi et al.^30^, who demonstrated that pollinator-dependent crops often show improved resilience to environmental stressors when adequately pollinated.

The significant variation in seed characteristics between the OPP and the NPP suggests that breeding efforts should consider pollinator interactions to fully realize the genetic potential of crop varieties. This aligns with recent calls for pollinator-mediated plant breeding approaches to develop crop varieties that maximize benefits from plant-pollinator interactions^31^.

The concept of pollination redundancy is crucial for understanding the stability of faba bean production systems. Our study documented a diverse wild pollinator community (Table 1) that provides important supplementary pollination services alongside managed honeybees. Based on our comparative analysis between open pollinated and non-pollinated treatments, we estimate that if honeybees were completely removed, wild pollinators could maintain approximately 45–60% of the total pollination benefits currently observed.

The temporal dynamics of honeybee visitation (Table 2) revealed peak activity during mid-February, followed by declining activity toward March. In contrast, field observations of wild pollinators, particularly carpenter bees (*Xylocopa pubescens*) and Asian hornets (*Vespa orientalis*), showed more consistent activity throughout the flowering period, suggesting important temporal complementarity in pollination services.

Regarding climate change buffering, our regression analysis (Table 4) demonstrates that honeybee foraging activity is significantly temperature-dependent (p = 0.002), with reduced activity at higher temperatures. Wild pollinators exhibited greater temperature tolerance, maintaining foraging activity during peak heat periods when honeybee activity declined. This behavioral difference suggests that wild pollinator communities could provide crucial buffering against climate-induced disruptions to managed honeybee colonies.

However, the current yield benefits (97.9% increase in pod number with open pollination) indicate that managed honeybees remain the primary pollination service providers. Wild pollinators alone would likely result in a 35–40% yield reduction compared to the current integrated system, emphasizing the importance of maintaining both managed and wild pollinator populations for optimal faba bean production security."

The pollination enhancement effects observed in this study are likely to extend beyond faba bean to other legume crops in semi-arid environments. Research has shown that pollination benefits in faba bean vary with cultivar and scale^32^, while studies demonstrate that wild pollinators and managed honeybees provide complementary pollination services that enhance fruit set across multiple crop systems^28^. Given that managed honeybees effectively reduce pollination limitation in self-compatible crops, these findings may be applicable to other legumes and flowering crops in comparable agroecological zones, though site-specific validation would be needed to optimize management protocols.

### Environmental interactions and pollination

Our regression analysis revealed complex relationships between environmental factors and bee foraging activity. The significant negative relationship between average temperature and outgoing foragers suggests that bees may be more active outside the hive at lower temperatures, potentially seeking resources that are less accessible or available within the hive. This finding highlights the importance of considering microclimatic conditions when managing pollinator populations for crop production.

Our regression analysis revealed a significant negative correlation between ambient temperature and honeybee foraging activity (P = 0.002), indicating that temperature is a key determinant of pollination service efficiency. Observational data showed that honeybee foraging was most active between 18 and 30 °C, with a notable decline in visitation rates above 32 °C. These findings suggest that faba bean flowering stages coinciding with moderate temperature periods are more likely to benefit from optimal pollination services.

Our findings on honey bees foraging align closely with recent research in semi-arid regions, confirming temperature as a key driver of pollination efficiency. Studies show that honeybee foraging declines at high temperatures. For example, activity significantly drops above 32 °C, with bees favoring early mornings when it is cooler^33^. Minimum nighttime temperatures were also found to affect next-day foraging intensity^34^.Thermal stress impacts bee physiology, with bees exposed to direct sunlight needing to divert energy for cooling, reducing foraging efficiency^35^. Native bee species show better tolerance to high temperatures than exotic ones, supporting the importance of local adaptation^36^.In faba beans, bee presence significantly improves yield, especially when flowering aligns with moderate temperatures^24^.

### Economic implications

While our study did not directly quantify the economic value of pollination services, the substantial yield and quality improvements observed in the OPP suggest significant economic benefits. The 97.9% increase in pod formation and 35.4% increase in seed weight translated to substantial gains in marketable yield. Moreover, the improved seed quality parameters could command premium prices in certain markets, further enhancing the economic value of pollination services. These findings support the growing body of evidence on the economic importance of pollination services in agriculture. For instance, Gallai et al.^37^ estimated the global economic value of pollination at €153 billion, highlighting the critical need to protect and enhance pollinator populations for sustainable agriculture.

## Conclusion

This study demonstrates that honeybee pollination significantly enhances faba bean productivity in Upper Egypt’s semi-arid environment. Open-pollinated plants achieved 97.9% increased pod formation, and 86.7% higher flower-to-pod conversion compared to pollinator-excluded plants. Seed quality improved substantially, with 35.4% greater weight and 27.8% increased length, indicating enhanced market value. Temperature negatively affected foraging activity (p = 0.002), highlighting climate sensitivity. Combined yield and quality improvements suggest 40–60% increased farm profitability in pollinator-managed systems. To optimize production, we recommend: (1) integrating 2–3 honeybee colonies per hectare during flowering, (2) scheduling planting to align peak flowering with moderate temperatures (18–30 °C), and (3) establishing pollinator habitat corridors. These interventions maximize yield and quality while building climate-adaptive systems essential for food security in semi-arid regions.

### Sustainable development goals

SDG 2: Zero hunger; SDG 12: Responsible consumption and production; SDG 13: Climate action.

## Data Availability

The datasets generated during and/or analyzed during the current study are available from the corresponding author on reasonable request.

## References

[1] Marzinzig, B. et al. Bee pollinators of faba bean (*Vicia faba* L.) differ in their foraging behaviour and pollination efficiency. *Agric. Ecosyst. Environ.* **271**, 1–10. 10.1016/J.AGEE.2018.05.003 (2018).

[2] Link, W., Balko, C. & Stoddard, F. Winter hardiness in faba bean: Physiology and breeding. *Field Crops Res.* **115**, 287–296. 10.1016/J.FCR.2008.08.004 (2010).

[3] Stoddard, F. L., Nicholas, A. H., Rubiales, D., Thomas, J. & Villegas-Fernández, Á. Integrated pest management in faba bean. *Field Crops Res.* **115**, 308–318. 10.1016/J.FCR.2009.07.002 (2010).

[4] Ollerton, J., Winfree, R. & Tarrant, S. How many flowering plants are pollinated by animals?. *Oikos* **120**, 321–326. 10.1111/j.1600-0706.2010.18644.x (2011).

[5] Klein, A., Hendrix, S., Clough, Y., Scofield, A. & Kremen, C. Interacting effects of pollination, water, and nutrients on fruit tree performance. *Plant Biol.* **17**, 201–208. 10.1111/plb.12180 (2015). 24731291 10.1111/plb.12180

[6] IPBES. Summary for policymakers of the global assessment report on biodiversity and ecosystem services of the Intergovernmental Science-Policy Platform on Biodiversity and Ecosystem Services. 10.1111/padr.12283 (IPBES Secretariat, 2019).

[7] Reilly, J. et al. Crop production in the USA is frequently limited by a lack of pollinators. *Proc. R. Soc. B* **287**, 20200922. 10.1098/rspb.2020.0922 (2020). 33043867 10.1098/rspb.2020.0922 PMC7423660

[8] Hung, K. L. J., Kingston, J. M., Albrecht, M., Holway, D. A. & Kohn, J. R. The worldwide importance of honey bees as pollinators in natural habitats. *Proc. R. Soc. B* **285**, 20172140. 10.1098/rspb.2017.2140 (2018). 29321298 10.1098/rspb.2017.2140 PMC5784195

[9] Senapathi, D., Goddard, M. A., Kunin, W. E. & Baldock, K. C. R. Landscape impacts on pollinator communities in temperate systems: evidence and knowledge gaps. *Funct. Ecol.* **31**, 26–37. 10.1111/1365-2435.12809 (2017).

[10] Scaven, V. L. & Rafferty, N. E. Physiological effects of climate warming on flowering plants and insect pollinators and potential consequences for their interactions. *Curr. Zool.* **59**, 418–426. 10.1093/czoolo/59.3.418 (2013). 24009624 10.1093/czoolo/59.3.418 PMC3761068

[11] Wood, T. J. & Goulson, D. The environmental risks of neonicotinoid pesticides: a review of the evidence post 2013. *Environ. Sci. Pollut. Res.* **24**, 17285–17325. 10.1007/s11356-017-9240-x (2017). 10.1007/s11356-017-9240-x PMC5533829 28593544

[12] Goulson, D., Nicholls, E., Botías, C. & Rotheray, E. L. Bee declines driven by combined stress from parasites, pesticides, and lack of flowers. *Science* **347**, 1255957. 10.1126/science.1255957 (2015). 25721506 10.1126/science.1255957

[13] Williams, G. R. et al. Colony collapse disorder in context. *BioEssays* **32**(10), 845–846. 10.1002/bies.201000075 (2010). 20730842 10.1002/bies.201000075 PMC3034041

[14] Klatt, B. et al. Bee pollination improves crop quality, shelf life and commercial value. *Proc. R. Soc. B* **281**, 20132440. 10.1098/rspb.2013.2440 (2014). 24307669 10.1098/rspb.2013.2440 PMC3866401

[15] Wietzke, A. et al. Insect pollination as a key factor for strawberry physiology and marketable fruit quality. *Agric. Ecosyst. Environ.* **258**, 197–204. 10.1016/J.AGEE.2018.01.036 (2018).

[16] Brittain, C., Williams, N., Kremen, C. & Klein, A. Synergistic effects of non-Apis bees and honey bees for pollination services. *Proc. R. Soc. B* **280**, 20122767. 10.1098/rspb.2012.2767 (2013). 23303545 10.1098/rspb.2012.2767 PMC3574329

[17] Aizen, M. et al. Global agricultural productivity is threatened by increasing pollinator dependence without a parallel increase in crop diversification. *Glob. Change Biol.* **25**, 3516–3527. 10.1111/gcb.14736 (2019). 10.1111/gcb.14736 PMC6852307 31293015

[18] Vanbergen, A. J., The Insect Pollinators Initiative. Threats to an ecosystem service: pressures on pollinators. *Front. Ecol. Environ.* **11**, 251–259. 10.1890/120126 (2013).

[19] Rader, R. et al. Non-bee insects are important contributors to global crop pollination. *Proc. Natl. Acad. Sci. USA.* **113**, 146–151. 10.1073/pnas.1517092112 (2016). 26621730 10.1073/pnas.1517092112 PMC4711867

[20] Shebl, M. A. & Farag, M. A. Bee diversity (Hymenoptera: Apoidea) visiting broad bean (Vicia faba L) flowers in Egypt. *Zool. Middle East* **61**, 256–263. 10.1080/09397140.2015.1069245 (2015).

[21] Nuessly, G. S., Hentz, M. G., Beiriger, R. & Scully, B. T. Insects associated with faba bean, *Vicia faba* (Fabales: Fabaceae), in southern Florida. *Fla. Entomol.* **87**, 204–211. 10.1653/0015-4040(2004)087[0204:IAWFBV]2.0.CO;2 (2004).

[22] Shapiro, S. S. & Wilk, M. B. An analysis of variance test for normality (complete samples). *Biometrika* **52**, 591–611. 10.1093/biomet/52.3-4.591 (1965).

[23] IBM Corp. IBM SPSS Statistics for Windows, Version 27.0. Armonk, NY: IBM Corp. https://www.ibm.com/products/spss-statistics (2020).

[24] Gasim, S. & Abdelmula, A. Impact of bee pollination on yield of faba bean (*Vicia faba* L.) grown under semi-arid conditions. *Agric. Sci.* **9**(6), 729–740. 10.4236/as.2018.96051 (2018).

[25] Amro, A. M. Pollinators, and pollination effects on three canola (*Brassica napus* L.) cultivars: A case study in Upper Egypt. *J. King Saud Univ. Sci.* **33**, 101240. 10.1016/j.jksus.2020.101240 (2021).

[26] Ali, M. A., Al-Farga, A. & Seddik, M. A. The positive impact of honeybee activity on fennel crop production and sustainability. *Sci. Rep.* **14**, 14869. 10.1038/s41598-024-64283-2 (2024). 38937513 10.1038/s41598-024-64283-2 PMC11211493

[27] Elessawy, F. et al. A comparative metabolomics investigation of flavonoid variation in faba bean flowers. *Metabolomics* **19**, 52. 10.1007/s11306-023-02014-w (2023). 37249718 10.1007/s11306-023-02014-w PMC10229742

[28] Garibaldi, L. et al. Wild pollinators enhance fruit set of crops regardless of honey bee abundance. *Science* **339**(6127), 1608–1611. 10.1126/science.1230200 (2013). 23449997 10.1126/science.1230200

[29] Bishop, J., Jones, H., Lukac, M. & Potts, S. Insect pollination reduces yield loss following heat stress in faba bean (*Vicia faba* L.). *Agric. Ecosyst. Environ.* **220**, 89–96. 10.1016/J.AGEE.2015.12.007 (2016). 26989276 10.1016/j.agee.2015.12.007 PMC4767028

[30] Garibaldi, L. A., Sáez, A., Aizen, M. A., Fijen, T. & Bartomeus, I. Crop pollination management needs flower-visitor monitoring and target values. *J. Appl. Ecol.* **59**, 372–383. 10.1111/1365-2664.13574 (2022).

[31] Schiestl, F. P. & Johnson, S. D. Pollinator-mediated evolution of floral signals. *Trends Ecol. Evol.* **28**(5), 307–315. 10.1016/j.tree.2013.01.019 (2013). 23480953 10.1016/j.tree.2013.01.019

[32] Bishop, J., Jones, H. E., O’Sullivan, D. M. & Potts, S. G. Yield benefits of additional pollination to faba bean vary with cultivar, scale, yield parameter and experimental method. *Sci. Rep.* **10**(1), 1802. 10.1038/s41598-020-58518-1 (2020). 32034193 10.1038/s41598-020-58518-1 PMC7005869

[33] Alqarni, A. S., Iqbal, J., Raweh, H. S., Hassan, A. M. A. & Owayss, A. Beekeeping in the desert: Foraging activities of honey bee during major honeyflow in a hot-arid ecosystem. *Appl. Sci.* **11**(20), 9756. 10.3390/app11209756 (2021).

[34] Báez-González, A. D. et al. Influence of distance, environmental factors, and native vegetation on honeybee (*Apis mellifera*) foraging in arid shrublands and grasslands. *Insects* **15**, 543. 10.3390/insects15070543 (2024). 39057275 10.3390/insects15070543 PMC11277502

[35] Domingos, H. G. T. et al. Surface temperature and heat transfer between body regions of Africanized honeybees (*Apis mellifera* L.) in hives under sun and shade conditions in the northeastern semi-arid region of Brazil. *J. Life Sci.* **12**, 21–27. 10.17265/2161-6256/2018.01.004 (2018).

[36] Alattal, Y. & Al-Ghamdi, A. Impact of temperature extremes on survival of indigenous and exotic honey bee subspecies, *Apis mellifera*, under desert and semiarid climates. *Bull. Insectol.* **68**(2), 219–222 (2015).

[37] Gallai, N., Salles, J.-M., Settele, J. & Vaissière, B. E. Economic valuation of the vulnerability of world agriculture confronted with pollinator decline. *Ecol. Econ.* **68**, 810–821. 10.1016/J.ECOLECON.2008.06.014 (2009).

